# Benign fibrous histiocytoma of larynx: A rare cause of acute airway obstruction

**DOI:** 10.4103/0970-2113.68318

**Published:** 2010

**Authors:** Shiv Sagar Gupta, Sanjay Singhal

**Affiliations:** *Department of TB and Chest Diseases, Era’s Lucknow Medical College, Lucknow, India*

**Keywords:** Airway, benign, histiocytoma, larynx

## Abstract

Fibrous histiocytoma of larynx is a rare entity. We herein present a 35-year-old femaleof benign fibrous histiocytoma oflarynx with severe airway obstruction that requiring urgent endotracheal intubation followed by tracheostomy. We also report the good long-term survival of this case after such a critical condition.

## INTRODUCTION

Fibrous histiocytoma is uncommon in the head and neck region and rare in the larynx.[1] Herein we present a case of fibrous histiocytoma of larynx with severe upper air way obstruction requiring emergent air way management.

## CASE REPORT

A 35-year-old female was admitted to the ICU of Era’s Lucknow medical college, in a gasping condition. On examination, she was cyanosed with cold peripheries. Her blood pressure was not recordable and on palpation, carotid pulse was very feeble. Arterial blood gas analysis revealed PaO _2_ 40.3 mm Hg, PaCO _2_ 85.4 mmHg and oxygen saturation of 64.7%. Endotracheal intubation was done with great difficulty because of huge supraglottic lump and very narrow glottic opening, with endotracheal tube size 6 mm and she was put on mechanical ventilation. Fluid resuscitation was given followed by nor-adrenaline infusion along with antibiotics and other supportive treatment.

**Figure 1:**
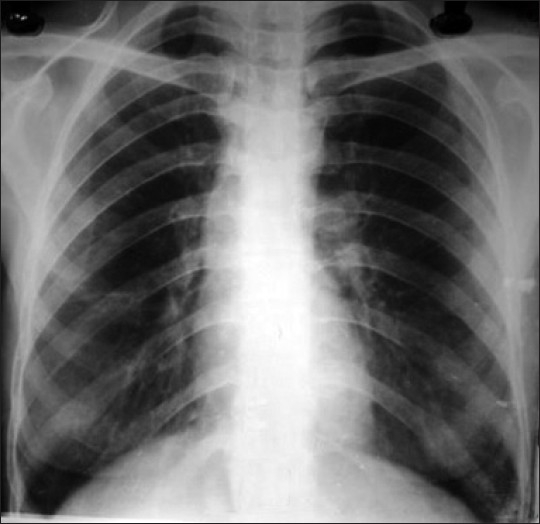
Normal chest radiograph

**Figure 2:**
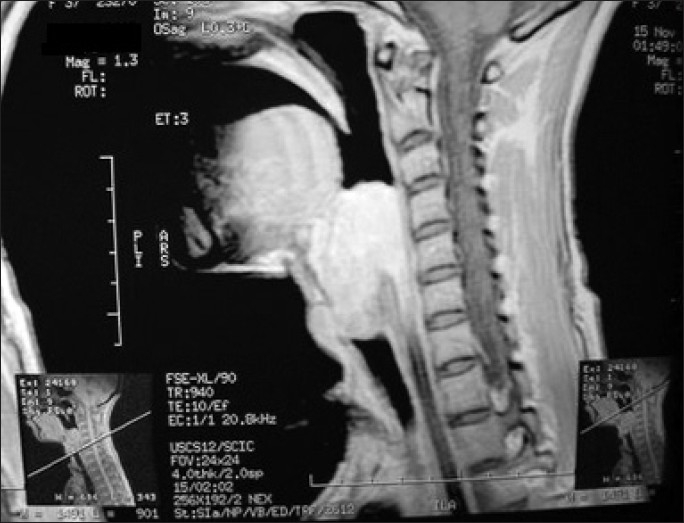
Computed tomography (CT) scan of neck revealing enhancing soft tissue mass starting below the vocal cord extended into the oropharynx (Sagital View)

**Figure 3:**
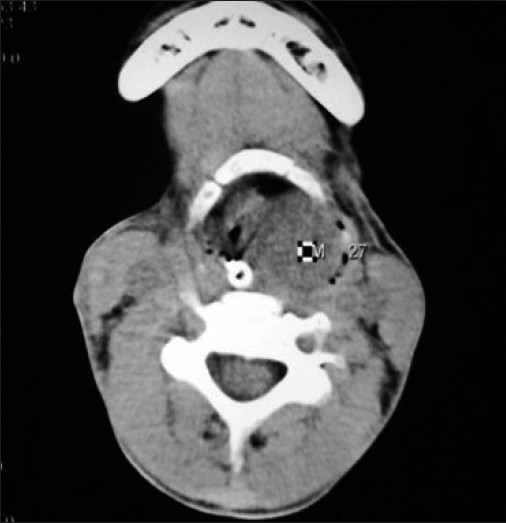
Computed tomography (CT) scan of neck revealing enhancing soft tissue mass predominantly on left side (Transverse View)

On detailed evaluation of previous records, she was having fever (low grade), cough, change in voice and breathing difficulties for last two months. She had consulted a local physician and he diagnosed her as a case of laryngeal tuberculosis based on history and positive mantoux test (indurations 12 mm). Previous chest radiograph revealed no abnormality[Figure 1]. Computed tomography (CT) scan of neck revealed enhancing soft tissue mass predominantly on left side starting below the vocal cord extended into the oropharynx [Figures2 and 3]. Then tracheostomy was done in view of supraglottic lump.

She gradually improved and weaned off from the mechanical ventilation and was referred to Sanjay Gandhi Institute of Medical Sciences for further management where biopsy was taken from the mass. The histopathological diagnosis of the biopsy was benign fibrous histiocytoma (BFH) of larynx. The patient underwent resection of the tumor by lateral pharyngeal approach. The general condition of the patient improved after the operation and she was discharged subsequently. For more than two years postoperatively, there has been no evidence of local recurrence.

## DISCUSSION

Fibrous histiocytoma usually occurs in the soft tissues, tendons and joints of the upper and lower extremities.[2] It is uncommon in the head and neck region[2] and rare in the larynx. It has been reported that biopsy specimens of malignant fibrous histiocytoma in the head and neck region were initially diagnosed as fibrosarcomas or osteosarcomas.[3–4] It is a mesenchymal tumor probably of histiocytic origin and may be divided into six subtypes,[2] i.e., pleomorphic, fibrous, giant cell, angiomatoid, myxoid and inflammatory, to be distinguished on the basis of the predominant feature. Surgery is the treatment of choice as radiotherapy and chemotherapy is not effective.[5–6] Excision with wide margins is necessary as tumors are characterized by a high rate of local recurrence varying from 44 to 73%.[7] On review of literature, this is the first case report of benign fibrous histiocytoma of larynx with such emergent condition from India. The present case implicates that acute upper airway obstruction can be the fatal presentation of fibrous histiocytoma requiring urgent management of airway.
